# The clinical significance of neutrophil gelatinase‐associated lipocalin in ischemic stroke patients with acute kidney injury

**DOI:** 10.1002/jcla.22907

**Published:** 2019-05-07

**Authors:** Wei Xiao, Wei Chen, Hanning Hu, Xiaomei Huang, Yi Luo

**Affiliations:** ^1^ Department of Nephrology, Tongji Medical College Wuhan Central Hospital, Huazhong University of Science and Technology Wuhan China; ^2^ Department of Laboratory Medicine Zhongnan Hospital of Wuhan University Wuhan China

**Keywords:** acute ischemic stroke, acute kidney injury, neutrophil gelatinase‐associated lipocalin

## Abstract

**Background:**

Acute kidney injury (AKI) has become a common complication of acute ischemic stroke (AIS) and may have a significant impact on the clinical outcomes. Neutrophil gelatinase‐associated lipocalin (NGAL), an acute phase protein, has been identified as a novel biomarker for acute kidney impairment. Here, we studied the early expression of NGAL in AIS patients with AKI and its clinic value in predicting and diagnosis of AKI after stroke.

**Methods:**

A total of 205 subjects diagnosed as first‐ever AIS were recruited in this study, including 40 AIS with AKI and 165 AIS without AKI defined using the KDIGO guidelines. The serum and urine levels of NGAL were measured with ELISA. To evaluate the clinic value of NGAL, we also detected creatinine, urea nitrogen and cystatin C and microalbumin (mALB) in serum or urine using chemiluminescence or immunoturbidimetry method, and then the receiver operating characteristic (ROC) curve and correlation was analyzed. The severity of AIS patients was evaluated based on the National Institutes of Health Stroke Scale (NIHSS) score.

**Results:**

The serum and urine NGAL levels were significantly increased in AIS patients with AKI, and line regression analysis indicated that there was a positive correlation between the serum NGAL and creatinine level in AIS patients accompanied AKI. Additionally, the concentration of serum NGAL in AIS patients with AKI increased with the severity of stroke.

**Conclusion:**

The increased serum NGAL may be used as a valuable complementary marker for the diagnoses and prediction of AKI in the early stage of AIS patients.

## 1 INTRODUCTION

Neutrophil gelatinase‐associated lipocalin (NGAL), also known as apolipoprotein‐2 or ferritin, is a member of the apolipoprotein superfamily.^1^, ^2^ Under healthy conditions in human and rodent, NGAL has been identified with cell‐specific expression in several tissues like kidney, lung, liver, spleen, heart, brain, and pancreas.^3^ During the inflammation, tissue damage, or stress, NGAL may act as an acute phase protein and its level could be upregulated in various cell types such as neutrophils, renal tubular epithelial cells, alveolar macrophages, and so on.^4^ Recent studies have suggested that serum or urine NGAL may be a useful biomarker in several pathological conditions including kidney diseases, tumors, and cardiovascular diseases like heart failure and diabetes.^5^, ^6^, ^7^, ^8^ Especially after primary or secondary renal injury, the elevated NGAL in urine or blood is accepted as an independent marker predicting severity and outcome of acute kidney injured patients.^9^

The expression and localization of NGAL in human brain tissue have been detected, and NGAL was mainly expressed and located in neurons, which indicated that NGAL may have central nervous system (CNS)‐specific functions.^10^, ^11^ Under conditions such as experimental autoimmune encephalomyelitis (EAE), traumatic brain injury (TBI), and cerebral aneurysms, the expression and activity of NGAL was increased.^10^, ^12^, ^13^ Elevated urinary NGAL levels were also found to be an early marker of acute kidney injury (AKI) in patients with TBI.^14^ Acute ischemic stroke (AIS) is a leading cause of death and disability worldwide, which may induce an acute impairment of kidney function in clinic because of various factors, such as renal infarction, high blood pressure, hyperglycemia, and infectious complications.^15^ Thus, AKI is not so rare in AIS patients, which may be an important factor affecting clinical outcomes. As a sensitive index for renal injury, whether NGAL could be an early biomarker of AKI in patients with AIS remains poorly understood.

In this study, we enrolled 205 AIS patients and 80 controls from Zhongnan Hospital of Wuhan University with the purpose of investigating the expression pattern of serum and urine NGAL and its clinical significance in early diagnosis and prediction of AKI after ischemic stroke.

## 2 MATERIALS AND METHODS

### 2.1 Clinical subjects

In this cohort study, the 205 patients with AIS were recruited from October 2017 to March 2018 in the Department of Neurology of Zhongnan Hospital of Wuhan University. AIS was defined as a sudden onset of nonconvulsive and focal neurological deficit and was further classified into different subtypes on the basis of the diagnosis criteria of the Trial of Org 10172 in Acute Stroke Treatment study.^16^ Briefly, the inclusion criteria were clinical presentation of first‐ever stroke, age 18 years or older and diagnosed as acute cerebral infarction by means of brain imaging. Eighty patients diagnosed without AIS were selected as the control group. Patients should be excluded with the following conditions: cerebral hemorrhage, subdural hematoma, intracranial space‐occupying lesions, traumatic cerebrovascular damage, heart failure, malignant diseases, immunologic diseases, severe infection, or pregnancy. This study was approved by Medicine Research Ethics Committee of Zhongnan Hospital of Wuhan University, and all stroke and control patients gave informed consent before enrollment.

### 2.2 Clinical assessment

The medical information of these patients was collected, including age, sex, risk factors, common complications, standard laboratory tests, and previous medical history. Physical and neurological examinations, and cerebral CT or MRI were also performed. Neurological severity was assessed according to the National Institutes of Health Stroke Scale (NIHSS) score.^17^ Risk factors like hypertension, diabetes mellitus, hyperlipidemia, atrial fibrillation, and infectious complications were determined by the corresponding diagnostic criteria combined with medical history.

### 2.3 Definition of acute kidney injury

AKI was defined using the KDIGO guidelines previously described.^18^ In brief, occurring one of the following abnormalities within 14 days of the onset of the symptoms was regarded as indicating the presence of AKI: (a) an increase in serum creatinine (sCr) by ≥0.3 mg/dL (26.5 µmol/L) within 48 hours; (b) an increase in sCr to ≥1.5 times baseline within the previous 7 days; (c) Urine volume ≤0.5 mL/kg/h for 6 hours.

### 2.4 Sample collection and laboratory test

Blood samples were collected from each of the 205 fasting AIS patients within 48 hours of symptom onsets as well as from the 80 fasting control subjects. After clotting for 30 minutes at room temperature, the blood samples were centrifuged at 1600 × g for 10 minutes at 4°C. The samples were then divided into aliquots and stored at −20°C prior to the determination of creatinine, urea nitrogen, cystatin C, and NGAL. The urine samples were frozen immediately following collection, and stored at −20°C prior to analysis. For further analysis, the urine samples were thawed, divided into 10‐mL aliquots, and centrifuged at 1000 × g for 10 minutes at 4°C. The supernatant was collected and the levels of creatinine, urea nitrogen, microalbumin (mALB), and NGAL were determined.

Creatinine, urea nitrogen, and cystatin C in serum were tested on an Olympus AU5800 automatic biochemical analyzer (Beckman Coulter). Urine mALB was measured using the immunoturbidimetry method by specific protein analyzer (BN ProSpec, Siemens). The serum and urine levels of NGAL were quantified using human lipocalin‐2/NGAL Quantikine ELISA kit (R&D Systems) according to the manufacturer's instructions, with the intra‐assay and inter‐assay coefficients of variation (CV) for <10%. Briefly, diluted samples were added to the ELISA plate pre‐coated with capture antibody at 4°C for 2 hours on a shaking platform. Aspirated the contents of the microwells and washed for at least three times. Then the cold conjugate, substrate solution, and stop solution were added to each well step by step, and finally read the wells at 540 nm in a microplate reader (Multiskan FC; Thermo Scientific). The values of NGAL were expressed as ng/ml. All above assays were performed in triplicate and blinded manner at the Medicine Biochemistry Laboratory of Zhongnan Hospital.

### 2.5 Statistical analysis

SPSS 19.0 (SPSS) and GraphPad Prism 7.0 (GraphPad Software) were used for statistical analysis. Statistical differences among the different groups were assessed by one‐way ANOVA with SNK post‐test. Pearson analysis was used for the correlation analysis. The receiver operating characteristic (ROC) curve was done for measuring the sensitivity and specificity of serum and urine NGAL at different cut‐off values. All data were represented as mean ± standard deviation, and *P* < 0.05 was considered as statistical significance.

## 3 RESULTS

### 3.1 Clinical characteristics of enrolled AIS patients with AKI

AKI developed in 40 of 205 patients (19.5%) with acute ischemic stroke. The clinical background of the patients with and without AKI was shown in Table 1. The gender and age distribution did not differ significantly among the control group, AIS without and with AKI groups. According to the NIHSS score, the AIS patients with AKI had the higher median score than the AIS without AKI subjects, revealing the more severe AIS outcome. Hypertension and diabetes were more prevalent, and hyperlipidemia, atrial fibrillation, and infectious complications were less common in AIS patients who developed AKI. Compared to the normal subjects, the levels of serum creatinine and urea nitrogen were higher in AIS patients with AKI, but no significant difference between the control subjects and AIS patients without AKI. The serum cystatin C was not shown the obvious increase after AIS. Moreover, the positive rate of abnormal urinary mALB (*>*30 mg/L) was higher in AIS with AKI than that in AIS without AKI; however, in 15 of 40 AIS patients with AKI (37.5%), the level of urine mALB was still normal (*<*30 mg/L).

**Table 1:** Demographic and clinical characteristics of AIS patients and control subjects

Characteristic	Control	AIS with non‐AKI	AIS with AKI	*P*
Number of patients	80	165	40	
Male, n (%)	42 (52.5)	87 (52.7)	25 (62.5)	NS
Age, y ± SD	65.0 ± 8.7	66.8 ± 10.6	63.5 ± 9.0	NS
Median NIHSS score (IQR)		4 (2‐7)	7 (3‐11)	^#^
Risk factors, n (%)				
Hypertension		59 (35.8)	22 (55.0)	^#^
Diabetes mellitus		62 (37.6)	19 (47.5)	^#^
hyperlipidemia		44 (26.7)	11 (27.5)	
Atrial fibrillation		26 (15.8)	7 (17.5)	
Infectious complications, n (%)		33 (20.0)	9 (22.5)	
Laboratory tests (mean ± SD)				
Serum creatinine (µmol/L)	69.03 ± 11.02	73.29 ± 9.89	144.1 ± 26.67	*, ^#^
Serum urea nitrogen (mmol/L)	5.76 ± 1.62	5.82 ± 1.42	8.10 ± 2.29	*, ^#^
Serum cystatin C (mg/L)	0.77 ± 0.16	0.80 ± 0.14	0.80 ± 0.16	
Urine mALB >30 mg/L (n, %)	0, 0	10, 6.1	25, 62.5	*, ^#^

Note

Data reported as number (%), mean ± standard deviation (SD) or median (IQR). Abbreviations: AIS: acute ischemic stroke, AKI: acute kidney injury, IQR: interquartile range, mALB: microalbumin; NIHSS: National Institutes of Health Stroke Scale; NS: not significant (*P* > 0.05).

* Control vs AIS with AKI, *P* < 0.05.

^#^ AIS with AKI vs AIS without AKI, *P* < 0.05.

### 3.2 NGAL expression was elevated in serum and urine of AIS patients with AKI

**Figure 1:**
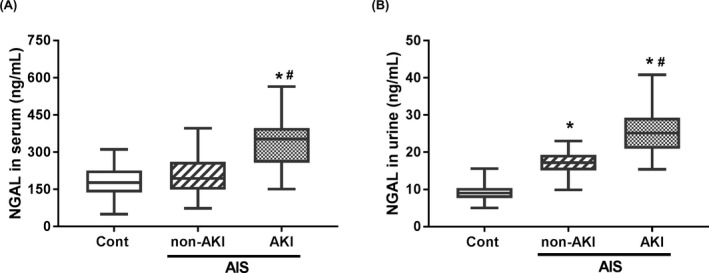
Serum and urinary NGAL levels in AIS patients with AKI. (A) Serum levels of NGAL in the control subjects (n = 80), AIS without AKI (n = 165), and AIS with AKI groups (n = 40). (B) Urinary levels of NGAL in the control subjects (n = 80), AIS without AKI (n = 165), and AIS with AKI groups (n = 40). * *P* < 0.05, compared with the control group; # *P* < 0.05, compared with the AIS without AKI group

In order to address the early expression features of NGAL in AIS patients with AKI within 48 hours of symptom onsets, serum and urine samples of all enrolled patients and healthy controls were collected and NGAL levels were measured by ELISA. Our data demonstrated that serum NGAL in AIS patients with AKI was much higher than health controls and AIS patients without AKI (Figure 1A). For urine NGAL, the levels were increased in AIS patients with or without AKI than control group, and the NGAL level in AIS patients with AKI was higher than that in AIS with non‐AKI (Figure 1B). These data suggested that serum and urine NGAL expression were both significantly increased on the early stage of AKI after AIS.

### 3.3 High expression of NGAL positively correlated with AKI severity following stroke

**Figure 2:**
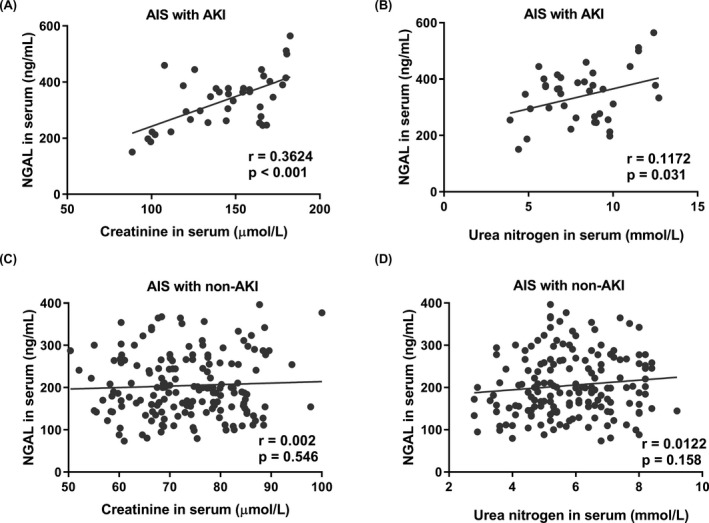
Correlation between serum NGAL level and the renal injury parameters in AIS patients with or without AKI. (A) Serum creatinine and (B) serum urea nitrogen in AIS patients with AKI (n = 40); (C) serum creatinine and (D) serum urea nitrogen in AIS patients with non‐AKI (n = 165)

**Figure 3:**
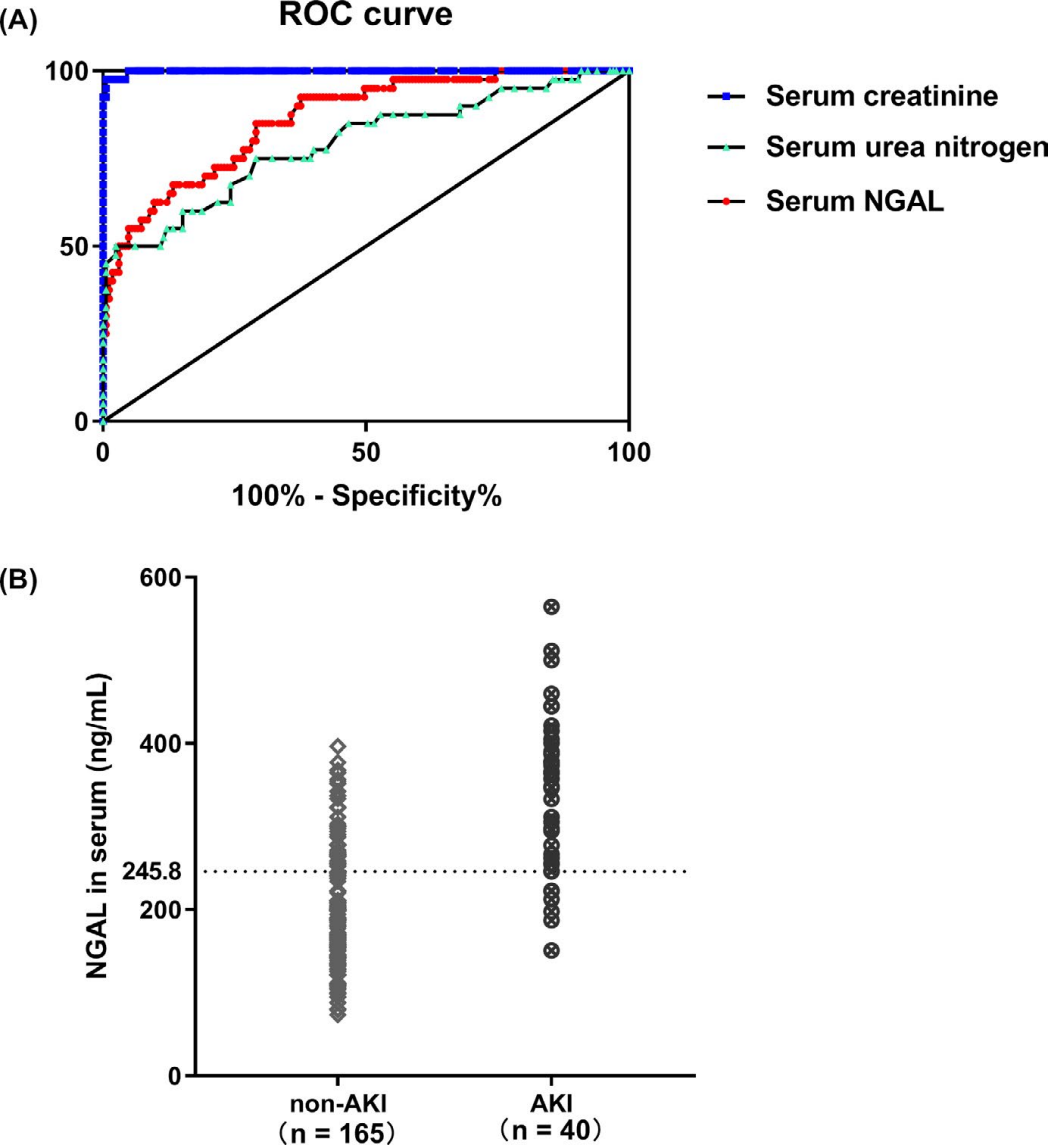
Receiver operating characteristic (ROC) curve and cut‐off value of serum NGAL in AIS with AKI group. (A) The area under ROC curve was 0.8639 for serum NGAL, 0.9986 for serum creatinine, and 0.7930 for urea nitrogen. (B) The cut‐off value of NGAL in serum was set at 245.8 ng/mL with sensitivity at 85% and specificity at 70.9%

KDIGO has suggested that AKI should be staged according to serum creatinine or urine output, which can reflect the injury severity.^18^ Here, correlation coefficients were calculated with the purpose to access the association between AKI severity and the NGAL level. The serum creatinine was recorded at admission to evaluate the kidney injury severity. Interestingly, there was a significant positive correlation between the serum NGAL and creatinine in the AKI following stroke (Figure 2A). The weak correlation was also found between the serum NGAL and urea nitrogen (Figure 2B). In the AIS patients with non‐AKI, there was no obvious correlation between the serum NGAL and creatinine or urea nitrogen (Figure 2C, 2D). Compared with the area under the ROC curve of serum creatinine (0.9986) and urea nitrogen (0.7930), it was 0.8639 for serum NGAL (Figure 3A). The cut‐off value of 245.8 ng/mL yield good sensitivity at 85% and specificity at 70.9% (Figure 3B). Therefore, higher expression of serum NGAL in the early stage of AIS may reflect the occurrence of AKI for emergent operation in clinic.

### 3.4 Serum NGAL positively correlated with NIHSS score in AIS patients with AKI

**Figure 4:**
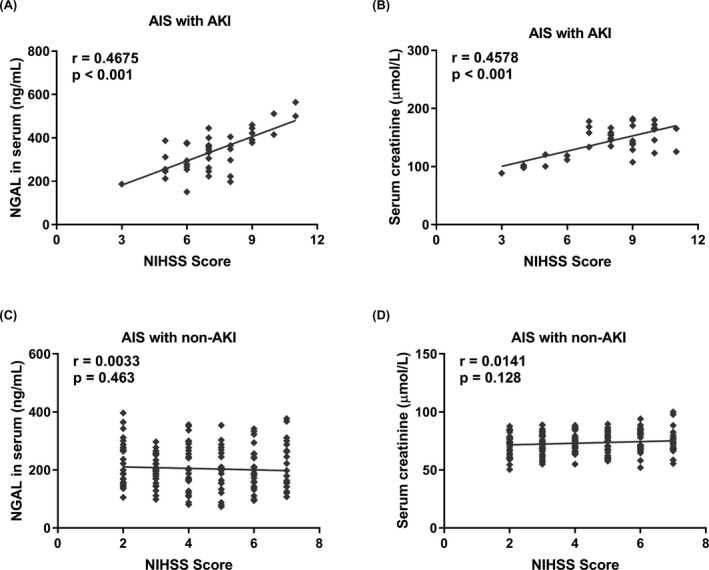
Correlation between serum NGAL or creatinine and the NIHSS score in AIS patients with or without AKI. (A) Serum creatinine and (B) serum urea nitrogen in AIS patients with AKI (n = 40); (C) serum creatinine and (D) serum urea nitrogen in AIS patients with non‐AKI (n = 165)

As shown in Table 1, the AIS patients with AKI had the higher median NIHSS score. To determine whether AKI may influence the clinical outcomes after stroke, we analyzed the relationship between serum creatinine or serum NGAL and severity of AIS. The linear regression analysis showed that the serum level of creatinine was found to correlate positively with NIHSS scores in AIS patients with AKI, and the similar correlation between serum NGAL and the NIHSS score also suggested that the levels of serum NGAL and creatinine may reflect the severity of AIS patients with AKI (Figure 4A, 4B). In the AIS patients with non‐AKI, no positive correlation was shown between the serum NGAL or creatinine and NIHSS score (Figure 4C,4D).

## 4 DISCUSSION

The present study investigated the change of serum and urine NGAL levels in AIS patients accompanied by AKI within 48 hours after onset. Increased serum NGAL was found in AIS patients with AKI, which was correlated positively with the value of serum creatinine. This finding suggests that serum NGAL level at the acute phase of AIS may be considered as a useful marker for diagnosis and prognosis of AKI in stroke.

In clinic, the diagnosis of AKI is still based on the detection of serum creatinine and urine volume.^19^ Other common indexes include urea nitrogen, cystatin C, urine mALB, glomerular filtration rate (GFR), and so on. However, detecting serum creatinine or urea nitrogen during the acute changes in renal function was shown not so reliable, which may reflect the kidney status accurately until the renal function reduced to a certain extent.^20^ Cystatin C is also an early marker of AKI, but it is difficult to be detectable in urine. Moreover, the calculation of GFR is also limited in clinic due to oliguria or other disturbance factors. Recently, NGAL as a novel marker for renal injury has been suggested to be an excellent predictor of AKI in several pathological conditions like circulatory shock, cirrhosis, and sepsis.^21^, ^22^, ^23^ The novel role of NGAL in CNS diseases such as EAE, TBI, and cerebral aneurysms has been highlighted gradually.^10^, ^12^, ^13^ Here, we found the high level of serum NGAL occurred in the AIS patients with AKI and its level was correlated positively with the level of serum creatinine, suggesting that serum NGAL may reflect the change and severity of renal function in the early stage of AIS. In AIS patients accompanied with AKI, hypertension and diabetes were common complications, which may be the main risk factors for the impairment of renal function after stroke. Additionally, studies have shown that significant changes of NGAL level can be detected using only 1 µL urine after induced kidney damage in mouse.^24^ We found that the levels of urine NGAL were both increased in AIS patients with or without AKI; however, the urinary mALB was still normal in some AIS patients with AKI. Thus, we speculate that urinary NGAL may be more sensitive than serum NGAL for predicting brain injury in an acute cerebrovascular event. Animal studies have demonstrated that cerebral inherent cells like astrocytes and endothelial cells can express NGAL.^25^, ^26^ The significant increase of urine NGAL in AIS patients with non‐AKI may reflect the ischemic brain damage, which need further elucidate. Acute kidney injury causes increased expression of NGAL mRNA, and the newly formed NGAL protein is released into the circulating blood and represents the majority of the “NGAL system pool”.^27^ Another part of systemic NGAL may come from the ischemic brain tissues via blood‐brain barrier (BBB) leakage. The significant increase of serum NGAL in AIS patients with AKI may not only reflect the damage of renal function, so our results did not show the diagnostic value of serum NGAL superior to that of serum creatinine. We also compared the clinic value of serum NGAL with serum creatinine and urea nitrogen according to the ROC curve analysis, and the results showed that the area under the curve in serum NGAL was 0.8639, which was fairly close to 1, indicating a better diagnostic value than serum urea nitrogen. A cut‐off value of 245.8 ng/mL yielded good sensitivity at 85% and specificity at 70.9%. The combined detection of serum creatinine and serum/urine NGAL in the AIS patients should be helpful for the prediction and diagnosis of acute kidney injury.

## 5 CONCLUSION

Serum NGAL may be used for the prognosis of the clinical outcome in the AIS patients with AKI, as higher serum NGAL was accompanied with the higher NIHSS score, showing the worse prognosis. Taken together, our data suggest that the increased serum NGAL may be a sensitive marker in response to acute renal injury after stroke, which can be valuable as a complementary and alternative biomarker for predicting and diagnosis of AKI following stroke. Furthermore, there were still some limitations in our study, and additional larger sizes of AIS patients with AKI from multiple centers are needed to validate our findings.

## References

[1] Kieldsen L , Bainton DF , Sengelov H , Borregaard N . Identification of neutrophil gelatinase‐associated lipocalin as a novel matrix protein of specific granules in human neutrophils. Blood. 1994;83(3):799‐807. 8298140

[2] Kieldsen L , Cowland JB , Borregaard N . Human neutrophil gelatinase‐associated lipocalin and homologous proteins in rat and mouse. Biochim Biophys Acta. 2000;1482(1–2):272‐283. 11058768 10.1016/s0167-4838(00)00152-7

[3] Friedl A , Stoesz SP , Buckley P , Gould MN . Neutrophil gelatinase‐associated lipocalin in normal and neoplastic human tissues. Cell type‐specific pattern of expression. Histochem J. 1999;31(7):433‐441. 10475571 10.1023/a:1003708808934

[4] Asimakopoulou A , Borkham‐Kamphorst E , Tacke F , Weiskirchen R . Lipocalin‐2 (NGAL/LCN), a “help‐me” signal in organ inflammation. Hepatology. 2016;63(2):669‐671. 26054053 10.1002/hep.27930

[5] Endre ZH , Westhuyzen J . Early detection of acute kidney injury: emerging new biomarkers. Nephrology. 2008;13(2):91‐98. 18275495 10.1111/j.1440-1797.2007.00905.x

[6] Candido S , Maestro R , Polesel J , et al. Roles of neutrophil gelatinase‐associated lipocalin (NGAL) in human cancer. Oncotarget. 2014;5(6):1576‐1594. 24742531 10.18632/oncotarget.1738 PMC4039233

[7] Kirbis S , Gorenjak M , Sinkovic A . The role of urine neutrophil gelatinase‐associated lipocalin (NGAL) in acute heart failure in patients with ST‐elevation myocardial infarction. BMC Cardiovasc Disord. 2015;15:49. 26070595 10.1186/s12872-015-0054-9 PMC4465307

[8] Vijay S , Hamide A , Senthikumar GP , Mehalingam V . Utility of urinary biomarkers as a diagnostic tool for early diabetic nephropathy in patient with type 2 diabetes mellitus. Diabetes Metab Syndr. 2018;4021(18):30099‐30107. 10.1016/j.dsx.2018.04.017 29673928

[9] Devarajan P . Review: neutrophil gelatinase‐associated lipocalin: a troponin‐like biomarker for human acute kidney injury. Nephrology. 2010;15(4):419‐428. 20609093 10.1111/j.1440-1797.2010.01317.x

[10] Zhou J , Chen H , Zhang M , et al. Early expression of serum neutrophil gelatinase‐associated lipocalin (NGAL) is associated with neurological severity immediately after traumatic brain injury. J Neuro Sci. 2016;368:392‐398. 10.1016/j.jns.2016.07.060 27538670

[11] Park KP , Rosell A , Foerch C , et al. Plasma and brain matrix metalloproteinase‐9 after acute focal cerebral ischemia in rats. Stroke. 2009;40(8):2836‐2842. 19556529 10.1161/STROKEAHA.109.554824 PMC3712850

[12] Berard JL , Zarruk JG , Arbour N , et al. Lipocalin 2 is a novel immune mediator of experimental autoimmune encephalomyelitis pathogenesis and is modulated in multiple sclerosis. Glia. 2012;60(7):1145‐1159. 22499213 10.1002/glia.22342

[13] Serra R , Volpentesta G , Gallelli L , et al. Metalloproteinase‐9 and neutrophil gelatinase‐associated lipocalin plasma and tissue levels evaluation in middle cerebral artery aneurysms. Br J Neurosurg. 2014. [Epub ahead of print] 10.3109/02688697.2014.913777 24799278

[14] Li N , Zhao WG , Xu FL , Zhang WF , Gu WT . Neutrophil gelatinase‐associated lipocalin as an early marker of acute kidney injury in patients with traumatic brain injury. J Nephrol. 2013;26(6):1083‐1088. 24249209 10.5301/jn.5000282

[15] Kamouchi M , Sakai H , Kiyohara Y , Minematsu K , Hayashi K , Kitazono T . Acute kidney injury and edaravone in acute ischemic stroke: the Fukuoka stroke registry. J Stroke Cerebrovasc Dis. 2013;22(8):e470‐476. 23800495 10.1016/j.jstrokecerebrovasdis.2013.05.018

[16] Adams Jr HP , Bendixen BH , Kappelle LJ , et al. Classfication of subtype of acute ischemic stroke. Definition for use in a multicenter clinical trial. TOAST. Trial of Org 10172 in Acute Stroke Treatment. Stroke. 1993;24(1):35‐41. 7678184 10.1161/01.str.24.1.35

[17] Brott T , Adams Jr HP , Olinger CP , et al. Measurements of acute cerebral infarction: a clinical examination scale. Stroke. 1989;20:864‐870. 2749846 10.1161/01.str.20.7.864

[18] Khwaja A . KDIGO clinical practice guidelines for acute kidney injury. Nephron Clin Pract. 2012;120(4):c179‐184. 22890468 10.1159/000339789

[19] Mehta RL , Kellum JA , Shah SV , et al. Acute Kidney Injury Network. Acute kidney injury network: report of an initiative to improve outcomes in acute kidney injury. Crit Care. 2007;11(2):R31. 17331245 10.1186/cc5713 PMC2206446

[20] Mishra J , Dent C , Tarabishi R , et al. Neutrophil gelatinase‐associated lipocalin (NGAL) as a biomarker for acute renal injury after cardiac surgery. Lancet. 2005;365(9466):1231‐1238. 15811456 10.1016/S0140-6736(05)74811-X

[21] da Rocha EP , Yokota LG , Sampaio BM , et al. Urinary neutrophil gelatinase‐associated lipocalin is excellent predictor of acute kidney injury in septic elderly patients. Aging Dis. 2018;9(2):182‐191. 29896409 10.14336/AD.2017.0307 PMC5963341

[22] Hamdy HS , El‐Ray A , Salaheldin M , et al. Urinary neutrophil gelatinase‐associated lipocalin in cirrhotic patients with acute kidney injury. Ann Hepatol. 2018;17(4):624‐630. 29893703 10.5604/01.3001.0012.0931

[23] Shyam R , Patel ML , Sachan R , Kumar S , Pushkar DK . Role of urinary neutrophil gelatinase‐associated lipocalin as a biomarker of acute kidney injury in patients with circulatory shock. Indian J Crit Care Med. 2017;21(11):740‐745. 29279634 10.4103/ijccm.IJCCM_315_17 PMC5699001

[24] Devarajan P . Neutrophil gelatinase‐associated lipocalin (NGAL): a new marker of kidney disease. Scand J Clin Lab Invest Suppl. 2008;241:89‐94. 18569973 10.1080/00365510802150158 PMC2528839

[25] Eqashira Y , Hua Y , Keep RF , Xi G . Acute white matter injury after experimental subarachnoid hemorrhage: potential role of lipocalin 2. Stroke. 2014;45(7):2141‐2143. 24893611 10.1161/STROKEAHA.114.005307 PMC4074774

[26] Jin M , Kim JH , Jang E , et al. Lipocalin‐2 deficiency attenuates neuroinflammation and brain injury after transient middle cerebral artery occlusion in mice. J Cereb Blood Flow Metab. 2014;34(8):1306‐1314. 24780901 10.1038/jcbfm.2014.83 PMC4126090

[27] Helanova K , Spinar J , Parenica J . Diagnostic and prognostic utility of neutrophil gelatinase‐associated lipocalin (NGAL) in patients with cardiovascular disease–review. Kidney Blood Press Res. 2014;39(6):623‐629. 25531230 10.1159/000368474

